# Negative Schottky Barriers and Spin-Polarized Fermi Crossings at WSe_2_/NbSe_2_ Interfaces

**DOI:** 10.1021/acsnano.5c22009

**Published:** 2026-02-25

**Authors:** Oliver J. Clark, Anugrah Azhar, Thi-Hai-Yen Vu, Benjamin A. Chambers, Federico Mazzola, Sadhana Sridhar, Geetha Balakrishnan, Aaron Bostwick, Chris Jozwiak, Eli Rotenberg, Sarah L. Harmer, Mohammad Saeed Bahramy, Michael S. Fuhrer, Mark T. Edmonds

**Affiliations:** † School of Physics and Astronomy, 2541 Monash University, Clayton, Victoria 3168, Australia; ‡ Diamond Light Source, Harwell Science and Innovation Campus, Didcot OX11 0DE, U.K.; § Department of Physics and Astronomy, University of Manchester, Oxford Road, Manchester M13 9PL, U.K.; ∥ Physics Study Program, Faculty of Science and Technology, Syarif Hidayatullah State Islamic University Jakarta, Tangerang Selatan 15412, Indonesia; ⊥ Flinders Microscopy and Microanalysis, 1065 Flinders University, Bedford Park, Adelaide, South Australia 5042, Australia; # Department of Physics and Astronomy ‘Galileo Galilei’, University of Padova, Padova, Italy; ¶ CNR-SPIN UOS Napoli, Complesso Universitario di Monte Sant’Angelo, Via Cinthia, 80126 Napoli, Italy; ∇ Department of Physics, 2707 University of Warwick, Coventry CV4 7AL, U.K.; ○ Advanced Light Source, Lawrence Berkeley National Laboratory, Berkeley, California 94720, United States; ⧫ Institute for Nanoscale Science and Technology, 1065 Flinders University, Bedford Park, Adelaide, South Australia 5042, Australia; †† ARC Centre for Future Low Energy Electronics Technologies, 2541 Monash University, Clayton, Victoria 3168, Australia; ‡‡ Melbourne Centre for Nanofabrication, Victorian Node of the Australian National Fabrication Facility, ANFF-VIC Technology Fellow, Clayton, Victoria 3168, Australia

**Keywords:** 2D material heterostructures, 2D material interfaces, p-type contacts, transition metal dichalcogenides, density functional theory, angle-resolved photoemission spectroscopy

## Abstract

Discovering and engineering spin-polarized surface states in the electronic structures of condensed matter systems is a crucial first step in the development of spintronic devices, wherein spin-polarized bands crossing the Fermi level can facilitate information transfer. Here, through nanofocused angle-resolved photoemission spectroscopy (nano-ARPES) and density functional theory-based calculations, we show that the interface between monolayer WSe_2_ and metallic NbSe_2_ exhibits a negative Schottky barrier height of ∼ −30 meV: the K-point valleys of the semiconducting layer are shifted by ∼800 meV to produce a surface-localized Fermi surface populated only by spin-polarized charge carriers. By increasing the WSe_2_ thickness, the Fermi pockets can be moved from K to Γ, demonstrating tunability of novel semimetallic phases that exist atop a substrate additionally possessing charge density wave and superconducting phases. Together, this study provides a spectroscopic understanding into p-type, Schottky barrier-free interfaces, which are of urgent interest for bypassing the limitations of current-generation vertical field effect transistors, in addition to longer-term spintronics development.

## Graphical abstract

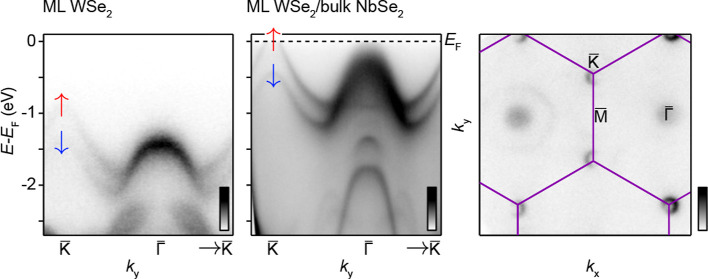

As the limitations of conventional electronics are reached, the semiconducting subsets of 2H-structured transition metal dichalcogenides (TMDs) have emerged as key components for ultraoptimized nanoscale electronics due to the ease with which their monolayer forms can be isolated without “dangling” bonds or residual polar charge. This facilitates, for example, the fabrication of vertical field effect transistors (VFETs) on the smallest possible length scales through interfacing with metallic 2D materials, like graphene.^1–4^ Despite this promise, most pairings of semiconducting TMDs with metals are found to exhibit large Schottky barriers, rendering them unsuited for applications due to suboptimal efficiency.^1^ Recent transport studies have identified WSe_2_/NbSe_2_ interfaces as a rare exception: the van der Waals nature of both materials precludes interface states from pinning the Fermi level to the band gap of the semiconductor, and the difference in work functions is compatible with the energetics of the WSe_2_ valence band maxima (VBM), together facilitating an ohmic contact.^2,3^ While these transport studies therefore demonstrate enormous potential for WSe_2_/NbSe_2_ systems, they do not rule out the presence of a small positive Schottky barrier at the interface. Furthermore, spectroscopic understanding of the momentum-resolved electronic structures at these interfaces is entirely lacking.

Here, through nano focused angle-resolved photoemission spectroscopy (nano-ARPES) and first-principles calculations, we examine the electronic structure at the interface between monolayer WSe_2_ and bulk NbSe_2_. We not only find a negative Schottky barrier but also the formation of a rich top layer-localized Fermiology exclusively composed of spin-polarized charge carriers. We further show how these Fermi surfaces can be tuned, both in terms of spin degeneracy and positioning in momentum space, and demonstrate how the key physics of these hybrid systems is robust against lattice mismatch, thus simplifying the assembly of these functional systems.

Together, this study provides a microscopic electronic structure perspective of p-type WSe_2_/NbSe_2_ interfaces while indicating potential ways to harness the unique spin-valley locked electronic structures of semiconducting 2H-TMDs at equilibrium.

## Results and Discussion

### Constructing WSe_2_/NbSe_2_ Interfaces

Figure 1a shows the electronic structure of monolayer WSe_2_ atop a weakly interacting graphite (gr) substrate. The band structure of 2H–WSe_2_, like other 2H-TMDs, naturally possesses pairs of spin–orbit split hole-like bands at the K and K′ points of its Brillouin zone (BZ). In the monolayer limit, these pairs of bands are spin-polarized out-of-plane due to an uncompensated in-plane electric dipole within a single 1H-unit^5–8^ but reverse from K to K′ with time-reversal symmetry (TRS). Together, this produces oppositely polarized valence band maxima (VBM) with maximal separation in momentum space.^5–8^ Electrons populating these Zeeman-like extrema at K and K′ can be selectively excited with circularly polarized light, which is directly exploited in so-called valleytronic devices.^5–7,9^ However, their energetic positioning of ∼ −0.75 eV below the Fermi level prevents contributions to transport without optical pumping or gating. Unlike the global VBM at K, the local valence band maxima (lVBM) at Γ is spin degenerate for all thicknesses, though due to significant interlayer hopping capability of the W d_z^2^_ orbitals from which it derives, it readily develops a large bandwidth along the *k*_*z*_ axis when neighboring layers are present.^10–14^

**Figure 1:**
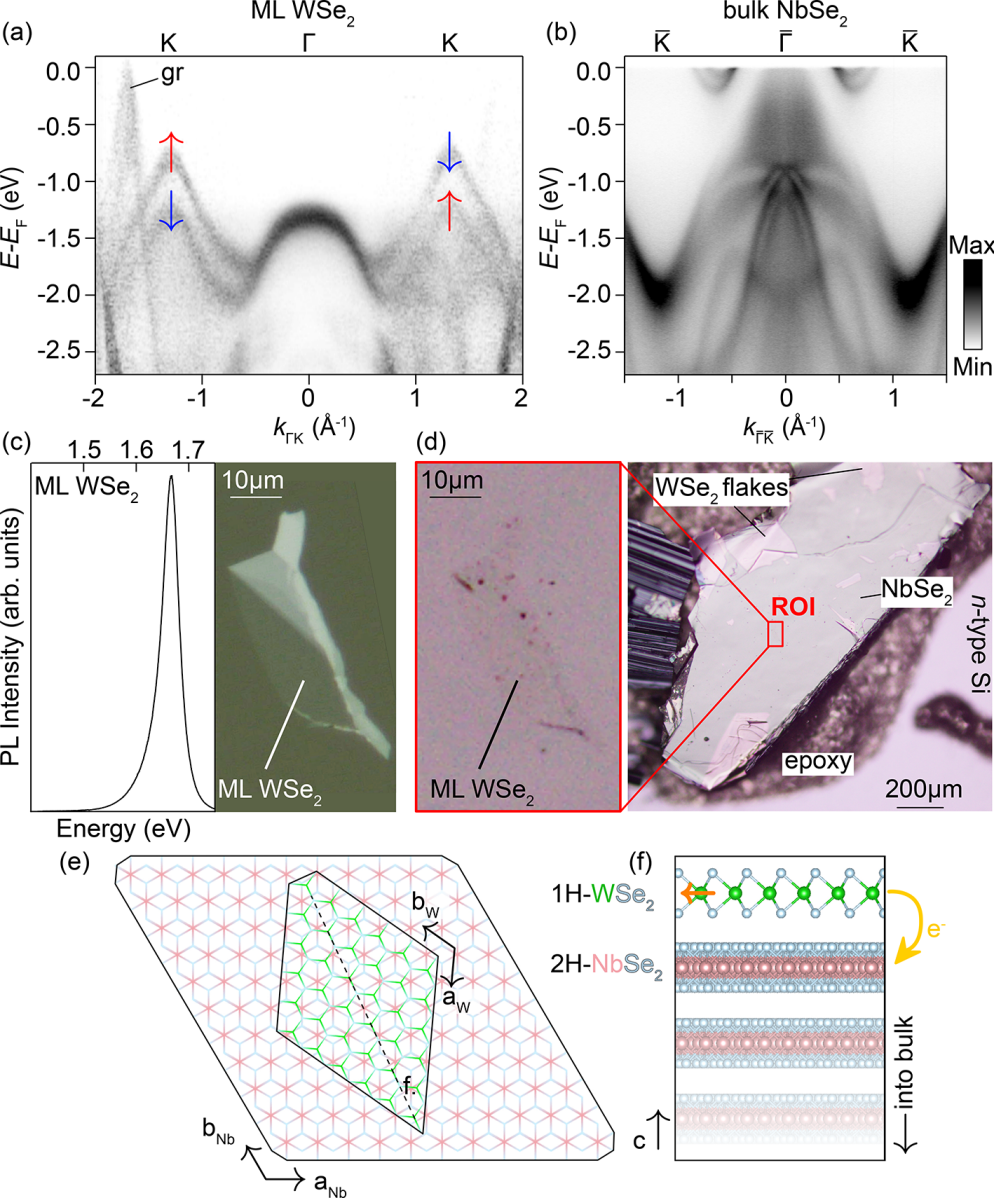
Electronic structures of ML WSe_2_ and bulk NbSe_2_, and heterostructure fabrication: (a) K′–Γ–K–band dispersion (hν = 21.2 eV, *T* = 300 K) of a monolayer of WSe_2_ deposited on a graphite/*n*-type Si substrate acquired with nano-ESCA. The out-of-plane spin polarization of the pair of spin–orbit split bands at K is shown, as determined in earlier works.^8^ The band labeled gr originates from the graphite Dirac cone of the substrate. (b) $\overset{®}{K^{\prime }\;}$ –Γ̅–K̅ band dispersions (*h*ν = 121 eV, *T* ∼10 K) of bulk 2H–NbSe_2_. (c) Optical image of the flake containing the ML-WSe_2_ region before transferring onto bulk NbSe_2_ (right) and photoluminescence (PL) signal from the monolayer region of the flake (left). (d) Optical image of a WSe_2_/(NbSe_2_)_*n*_ sample. The red box indicates the area where the WSe_2_ monolayer was transferred, shown left. (e,f) Schematic diagram of a monolayer of 2H–WSe_2_ placed upon bulk 2H–NbSe_2_ with a large twist angle. (*a*,*b*)_W,Nb_ are the crystallographic directions for WSe_2_ (W) or NbSe_2_ (Nb). The dashed line in the *a*-*b* plane in (e) indicates the cross section shown in (f). The orange arrow in the WSe_2_ layer indicates the electric dipole giving rise to the Zeeman-like out-of-plane spin polarization indicated in (a). The yellow curved arrow shows the direction of charge transfer in this hybrid system.

These band features are compared with those of bulk 2H–NbSe_2_, shown in Figure 1b. The crystal and electronic structures of 2H–WSe_2_ and 2H–NbSe_2_ are qualitatively very similar. However, the Nb d^1^ configuration (compared to W d^2^) positions the NbSe_2_ Fermi level below both Γ and K lVBM, creating a metallic Fermi surface with large electron and hole sheets.^15–17^ The low energy band structure at Γ̅ is therefore predominantly Nb d_z^2^_ and Se p_z_-derived and is again extremely three-dimensional due to the orbital hopping potential across the van der Waals gap, evident in photoemission spectra through the diffuse ‘filled-in’ spectral weight of the *k*_*z*_-projected band manifold at Γ̅ (Figure 1b). The electron-like sheets partway along the Γ̅–K̅ direction share the in-plane transition metal ( $d_{xy},d_{x^{2}-y^{2}}$ ) orbital character as the VBM of monolayer WSe_2_ and therefore remain layer-locked and two-dimensional even in the many-layer limit.^17^

Figure 1c–f provides an overview of the construction and composition of the hybrid system made by pairing a monolayer WSe_2_ flake to a bulk NbSe_2_ crystal. Flakes of WSe_2_ were exfoliated onto polydimethylsiloxane (PDMS) film, and the presence of monolayer regions was verified through their photoluminescence spectra (Figure 1c), which are strongly thickness-dependent for semiconducting 2H-TMDs.^18^ Flakes are then transferred onto cleaved surfaces of NbSe_2_ single crystals (Figure 1d) in an Ar glovebox. Despite the majority of the NbSe_2_ surface remaining exposed, the monolayer WSe_2_ cap is found to be sufficient to locally preserve the underlying substrate from oxidation for extended periods in atmospheric conditions. The crystal structure of the assembled WSe_2_/NbSe_2_ interface is shown in Figure 1e,f.

### Spin-Polarized Fermiology of 1 ML-WSe_2_/NbSe_2_

Figure 2a,b illustrates the loss of electrons from the WSe_2_ monolayer to the bulk NbSe_2_ crystal across the interface, underpinning the transport characteristics of these p-type contacts.^2,3^ The charge transfer is facilitated by the respective out-of-plane orbital-derived bands at Γ̅ in both materials, which readily hybridize when the constraints of 2D quantization are lifted. In contrast to previous reports, which could not rule out small positive Schottky barriers across similar WSe_2_/NbSe_2_ interfaces,^2,3^ our ARPES spectra in Figure 2c,f establish that the charge transfer from monolayer WSe_2_ to the bulk NbSe_2_ substrate is sufficient in scale to drive the formation of a semimetallic WSe_2_ ground state, thus implying a perfect ohmic contact between these two materials.

**Figure 2:**
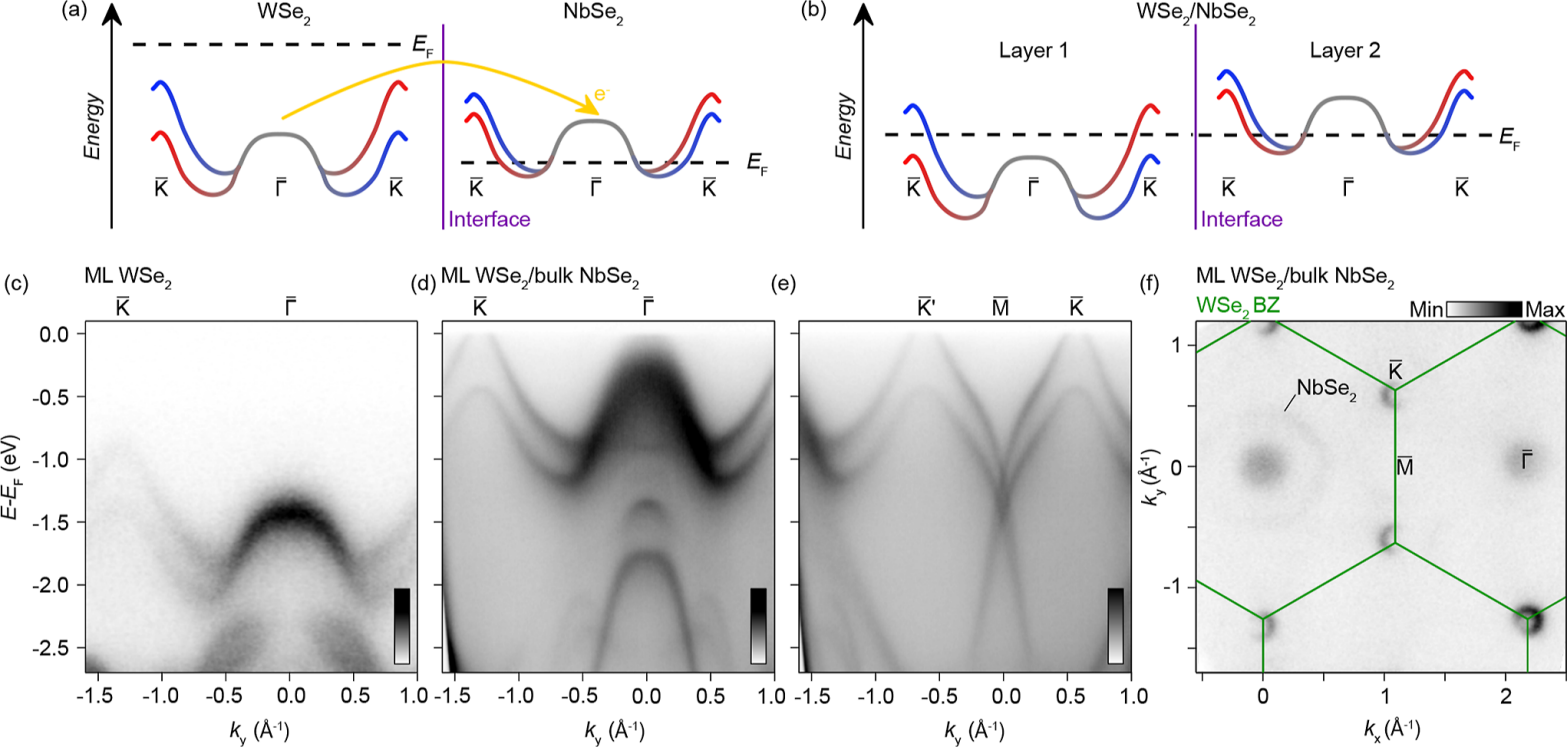
Fermiology of ML-WSe_2_ on bulk NbSe_2_: (a,b) Schematic diagrams of freestanding (a) and interfaced (b) WSe_2_ and NbSe_2_ systems. The band coloring indicates the out-of-plane spin polarization of the bands, though we note that for 2+ layer TMDs, the spin polarization is quenched by the adjacent layer in the unit cell. (c) Band dispersion (*h*ν = 114 eV) along the K̅–Γ̅–K̅ for a free-standing WSe_2_ monolayer, measured under similar experimental conditions to the heterostructure shown in (d). (d,e) Band dispersions (*h*ν = 91.5 eV) along the K̅–Γ̅–K̅ (d) and K̅–M̅–K̅ (e) directions of ML-WSe_2_ placed on a bulk NbSe_2_ substrate. (f) Corresponding Fermi surface (*h*ν = 91.5 eV). Solid lines show the BZ of WSe_2_.

Remarkably, the Fermi level is repositioned between the WSe_2_ lVBM at K and Γ to produce a top-layer electronic structure with momentum-separated Fermi pockets which are likely spin-polarized (as justified below). Band dispersions along the K̅–Γ̅–K̅ and K̅–M̅–K̅ directions in Figures 2d and 2e, respectively, show the full valence band structure of this charge-doped system. In comparison to a free-standing ML-WSe_2_ case (Figure 2c), while the d_z^2^_-derived lVBM at Γ is significantly broadened due to interactions with the out-of-plane orbital-derived bands of the substrate, the remainder of the electronic structure, derived from in-plane orbitals with negligible out-of-plane hopping, is relatively unchanged other than the Δ*E* ≈ 0.85 eV shift toward the Fermi level. This is calculated from the energy difference of the deeper-lying K-point valley between the data in Figures 1 and 2 and is found to be consistent across the interfaced region, as shown in Supplementary Figure 1. By assuming that the spin–orbit gap at K̅ remains unchanged, the binding energy of the K-lVBM above *E*_F_ can be extracted as the Schottky barrier height of ∼ −30 meV.

The corresponding Fermi surface of this hybrid system is shown in Figure 2f. The bands centered at K̅ derive from the shallower binding energy branch of the spin–orbit split valleys in monolayer WSe_2_, and the diffuse spectral weight at Γ̅ originates from the spectral tail of the broadened WSe_2_ Γ-lVBM. There are also larger features with low spectral weight centered at both the Γ̅ and M̅ points, which we attribute as signals directly from the substrate.^16,17^ We note that due to the surface sensitivity of ARPES necessitating unimpeded access to sample regions of interest, characterizing the electronic structure of a semimetallic phase of WSe_2_ without interfacing to NbSe_2_ would require levels of electrostatic doping beyond that possible either with, e.g., alkali metal deposition^19^ or through back gating exfoliated TMD flakes.^20^

### Orbital-Dependent Localization within WSe_2_/NbSe_2_ Heterostructures

In Figure 3, the contrasting dimensionalities of in- and out-of-plane orbital-derived bands (and therefore between the K̅ and Γ̅ lVBM, respectively) are explored through photon energy-dependent ARPES, which simultaneously varies both the probed *k*_*z*_ plane and the electron mean free path.^21^ K̅–Γ̅–K̅ band dispersions and Fermi surface maps for 121 and 150 eV photons, shown in Figure 3a,e and 3b,f, respectively, show increased spectral weight of bands imaged directly from the NbSe_2_ substrate relative to the datasets in Figure 2 (*h*ν = 91.5 eV) due to the increased effective transparency of the WSe_2_ top layer. The larger electron mean free path enables both direct confirmation of the preserved underlying NbSe_2_ band structure and clear visualization of a 24° relative twist angle between the substrate and the WSe_2_ top layer. An equivalent Fermi surface from a freshly cleaved NbSe_2_ crystal is presented in Supplementary Figure 2­(a) for comparison.

**Figure 3:**
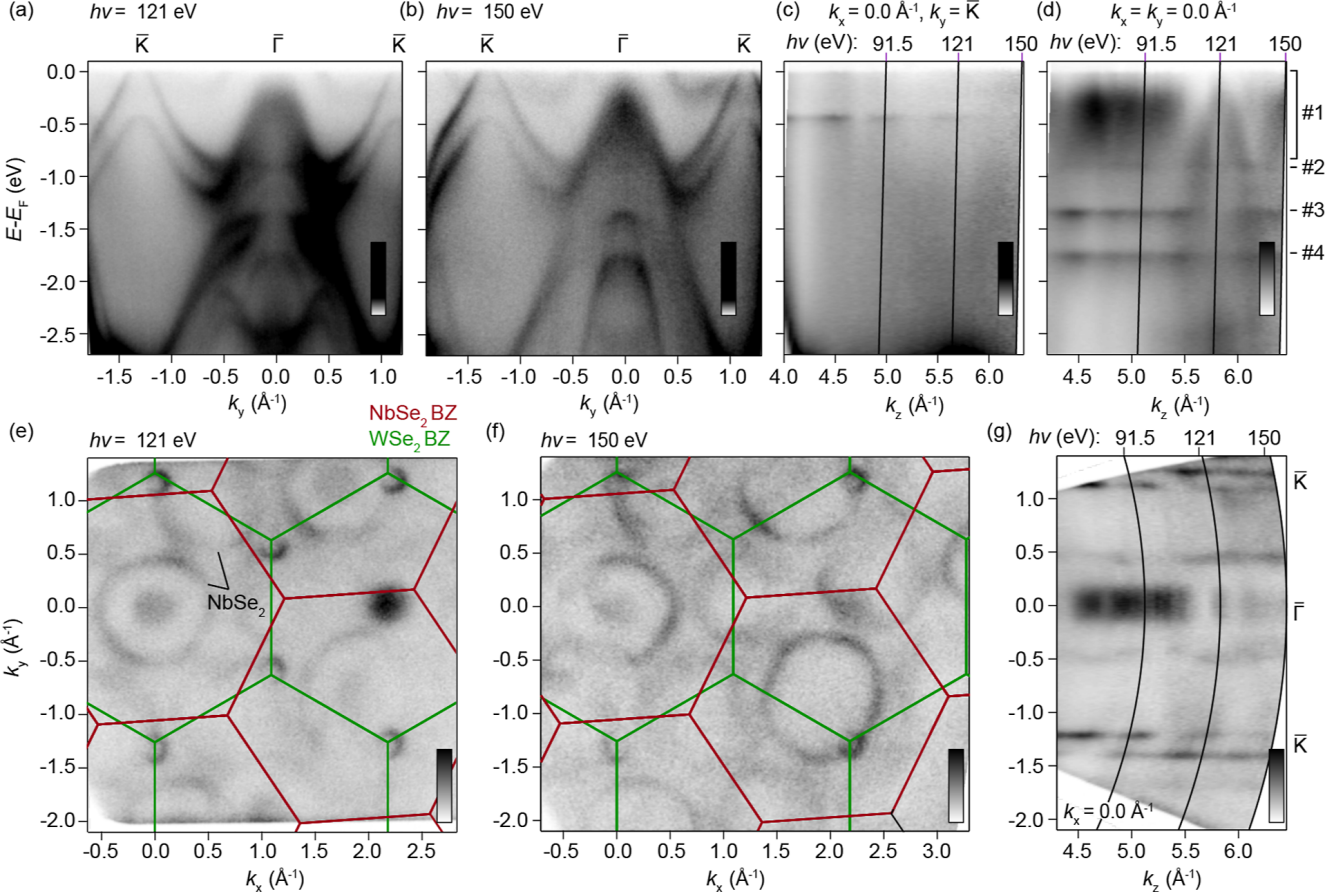
Mixed band dimensionality in ML-WSe_2_/bulk NbSe_2_: (a,b) Band dispersions along the K̅–Γ̅–K̅ directions for *h*ν = 121 eV (a) and (b) 150 eV. (c,d) *k*_*z*_ dispersions through the K̅ point (c) and Γ̅ point (d) constructed by varying the incident photon energy between 60 and 150 eV (*V*_0_ = 13 eV). (e,f) Fermi surface maps taken with *h*ν = 121 eV (e) and 150 eV (f). Red and green solid lines are Brillouin zone contours for NbSe_2_ and WSe_2_, respectively. (g) *k*_*y*_–*k*_*z*_ constant energy contour at the Fermi level constructed by varying the incident photon energy between 60 and 150 eV (*V*_0_ = 13 eV). Black lines in (c,d,g) indicate the *k*_*z*_ positioning of the 91.5 eV, 121 eV, and 150 eV data sets shown in Figures 2 and 3. The four distinct bands in (d) are labeled from low binding energy to high.

While the twist angle between the monolayer and bulk components of our heterostructures is large, we do not expect it to be consequential for charge transfer between these materials. As the orbitals with a non-negligible spatial extent into the van der Waals gap contribute bands primarily to the Γ̅ point in both WSe_2_ and NbSe_2_, interlayer coupling and band hybridization across the interface should be largely unhindered. Equivalently, the relevant hopping channels are available independently of twist angle.

The scale of this hybridization is shown in Figure 3d, where the *k*_*z*_ dependence of bands at the Γ̅ point is shown. There are four features within this energy window labeled from low to high binding energy: The shallowest band closest to the Fermi level (#1 in Figure 3d) is the lVBM of WSe_2_, which, as shown in Figures 2 and 3a,b, becomes significantly altered relative to the equivalent band in the free-standing case (Figure 1a and 2c). Its dispersive nature (most clearly resolved in the high *k*_*z*_ regime) directly evidences a delocalization along the *c*-axis into the bulk, thus permitting momentum dependence in the out-of-plane *k*_*z*_ direction. The other three bands are two-dimensional: the deepest bands at approximately −1.4 and −1.7 eV (#3 and #4 in Figure 3d) are mixed character Se p_xy_- and W d_xz,yz_-derived bands, which are unaffected by the details of the substrate due to the negligible *c*-axis hopping from in-plane orbitals.^8,15,22^ The remaining band is a sharp 2D state at −0.9 eV without a clear analogue in the noninteracting limit. While this band could be mistaken for a second *k*_*z*_ sub-band within the d_z^2^_ manifold present within bilayer WSe_2_, its two-dimensionality relative to the lower-energy band (#1) and the unambiguous photoluminescence signal from ML WSe_2_ before assembly (Figure 1c) mean that this is unlikely. Instead, we attribute this to be a signature of the bulk Dirac point and topological surface state pairs predicted in bulk 2H–NbSe_2_.^22^ Indeed, a Dirac-like band crossing is prominent in the data on bare bulk NbSe_2_ shown in Figure 1b and Supplementary Figure 2b,c, at the same binding energy and momentum.

Figure 3c shows an equivalent *k*_*z*_ dispersion for the K̅ point: in contrast to the Γ̅-lVBM, the pair of spin–orbit split bands at K̅ remain sharp, two-dimensional, and therefore monolayer-like as a function of *k*_*z*_, in line with previous discussions and the W $d_{xy,x^{2}-y^{2}}$ orbitals from which they derive. The contrasting dimensionalities of the lVBM are further seen in the *k*_*y*_ – *k*_*z*_ Fermi contour shown in Figure 3g, where bands near Γ̅ and K̅ are diffuse and well-defined, respectively. Note also that there is no evidence of band hybridization of the overlapping WSe_2_ and NbSe_2_ bands away from Γ̅ in the Fermi surfaces shown in Figure 3e,f, consistent with their in-plane orbital makeup and therefore their layer-locked localization. It follows that the spin structure of the observed K-point Fermi crossings should be identical to the equivalent bands of free-standing WSe_2_ monolayers, i.e., strongly spin-polarized along the out-of-plane direction.

In Figure 4, the experimentally observed orbital-dependent localization is further explored through comparisons to layer- and spin-resolved (⟨*S*_*z*_⟩) density functional theory-based calculations for a 1 ML-WSe_2_/5 ML-NbSe_2_ heterostructure with a zero twist angle. Despite the finite thickness of the NbSe_2_ component and the absence of a finite twist angle, the key physics of the experimental system is extremely well captured. The shallower binding-energy K point valleys of the WSe_2_ top layer cross the Fermi level to form a spin-polarized lVBM at ∼ −100 meV binding energy, reproducing the negative Schottky barrier seen experimentally. As demonstrated by their layer-resolved spectral weight, these strongly spin-polarized Fermi crossings are entirely absent away from the topmost layer, in line with the above discussions.

**Figure 4:**
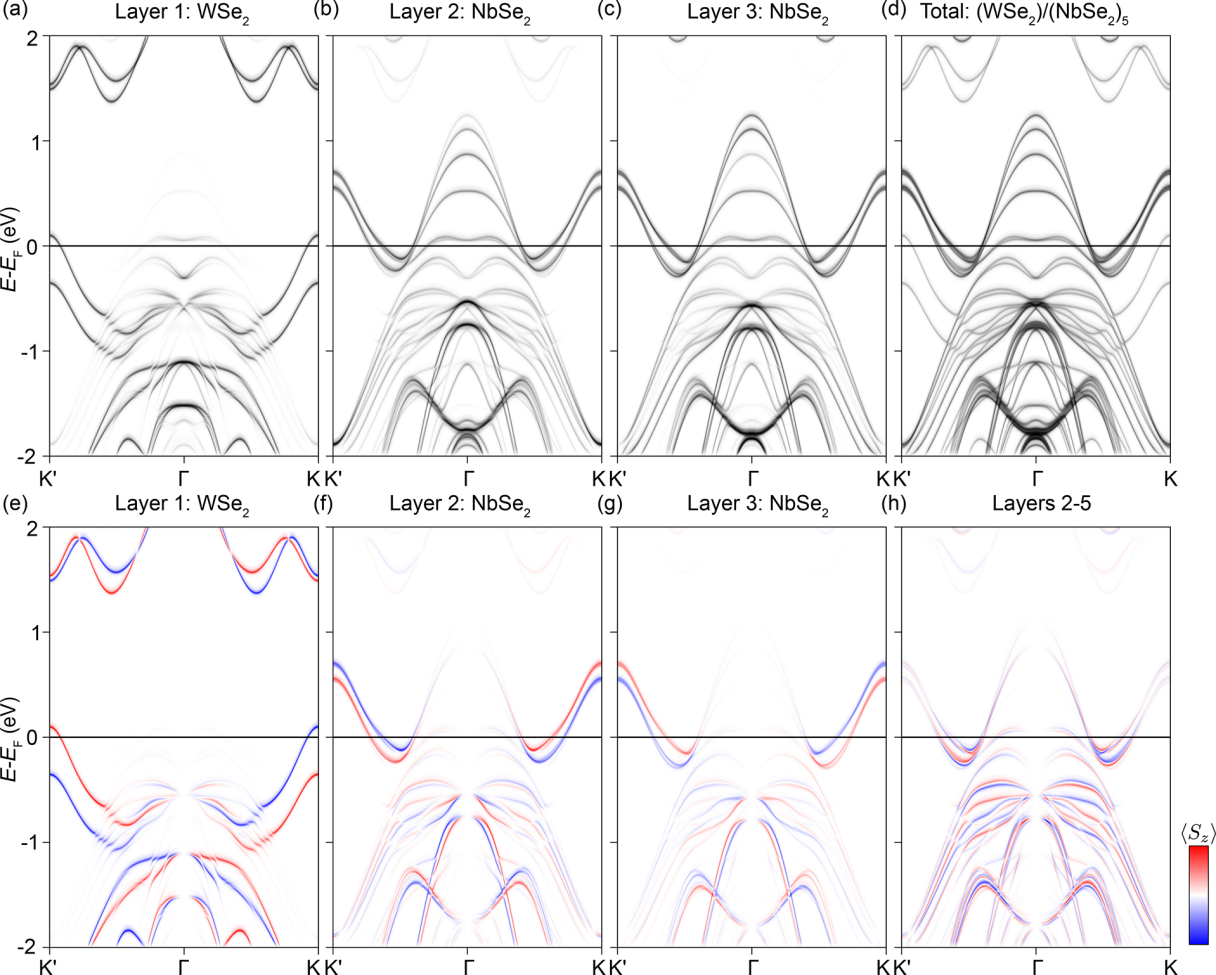
First-principles band structure calculations of a (WSe_2_/(NbSe_2_)_5_ heterostructure with a zero twist angle: (a–c) Layer-projected spectral function calculations to the first (a), second (b), and third (c) layers of this finite-size heterostructure. (d) Total layer-integrated spectral function calculation. (e–g) Corresponding ⟨*S*_*z*_⟩ spin projections for the spectral functions shown in (a–c). (h) ⟨*S*_*z*_⟩ contribution from the four NbSe_2_ layers closest to the surface (layers 2–5).

The contrasting behavior of the Γ̅-lVBM is also well-reproduced: as observed within the experimental band dispersions in Figures 2d and 3a,b,d, the Γ-lVBM of WSe_2_ becomes resonant with the equivalent bands in NbSe_2_ and is therefore delocalized through the heterostructure. The spectral weight of the collective d_z^2^_ manifold at Γ̅ is most prominent away from the surface (Figure 4b,c), with only very faint signatures in the WSe_2_ top layer. The surface transport channels from this hybrid system should therefore be dominated by bands at K̅ and will therefore be almost entirely spin-polarized.

We note that, while each individual NbSe_2_ layer also carries a significant, layer-localized spin polarization at K̅,^17^ the net spin polarization from centrosymmetric 2H–NbSe_2_ is zero due to the oppositely oriented electric dipoles between adjacent NbSe_2_ layers. This is shown in Figure 4h, where the total ⟨*S*_*z*_⟩ contribution from the four NbSe_2_ layers closest to the surface is significantly reduced relative to that of an individual layer. The bulk transport properties, therefore, will be dominated by spin-degenerate carriers from large hole bands at both Γ̅ and K̅ for layer 2+, with the heavily doped surface WSe_2_ monolayer being the only net contributor to the overall spin structure.

We stress that the strong agreement to the band structure calculations in Figure 4, despite the large experimental twist angle, is again strongly suggestive that both the degree of charge transfer and the resulting electronic structure are largely insensitive to the extent of the lattice mismatch between WSe_2_ and NbSe_2_, and therefore, the physics on show here can be exploited without complex assembly. This is consistent with the conclusion that interactions between out-of-plane orbital-derived states at Γ̅ are primarily responsible for facilitating charge transfer. However, we do not rule out smaller twist angle dependencies: While the Schottky barrier is negative in both theory and experiment, more direct Nb–W interlayer hopping pathways may slightly enhance charge transfer. Moreover, band hybridizations driven by Moiré periodicities may become apparent for specific twist angle regimes, as was found in numerous lattice-mismatched 2H-TMD bilayers.^23,24^

Taken together, the experimental and theoretical findings in Figures 3 and 4 evidence that the states at K̅ remain localized to the WSe_2_ top layer and can therefore be considered surface states of the hybrid bulk system. There has been significant effort in characterizing materials with large atomic spin–orbit coupling which are capable of hosting Rashba-split states either at the surface (due to the surface potential step) or within the bulk (due to noncentrosymmetric crystal structures) with a large band separation.^25–31^ The surface states of ML-WSe_2_/NbSe_2_ can be considered a particularly extreme example of this, with up and down spin species separated by the K–K′ separation of the BZ ( $\frac{4\pi }{3a}\approx$ 1.3 Å^–1^). The momentum separation of these states should provide significant resilience against interband scattering, similar to that found in metallic surface states of topological insulators (TIs).^32^

### Tunability through Thickness Augmentation

To finalize, we demonstrate how the broader physics of 2H–WSe_2_ is maintained despite the significant charge doping. The Γ̅ lVBM in free-standing WSe_2_ forms a dispersive band continuum along *k*_*z*_ in the bulk limit due to non-negligible out-of-plane hopping of the d_z^2^_ orbitals from which it derives. For intermediate thicknesses, it forms an array of energetically separated, doubly degenerate *k*_*z*_ subbands, the number of which matches the total thickness in monolayers. Since the details of the K point lVBM are relatively unaffected by the surrounding layers, the Γ̅-lVBM becomes the global valence band maximum for 3+ ML.^10–14^ In Figure 5, we show how this *c*-axis delocalization of out-of-plane orbital-derived electrons within the WSe_2_ top layers drastically alters the Fermiology of interfaced systems. In Figure 5a–f, ARPES spectra from 4 ML-WSe_2_, where the global valence band maximum is at the Γ point,^33–35^ and a 4 ML-WSe_2_/bulk NbSe_2_ system are compared. In analogy to the monolayer scenario, the charge transfer acts to shift the WSe_2_ bands toward the Fermi level by Δ*E* ≈ 0.63 eV, as calculated from the shift of the 2D state at Γ and ∼ −2.2 eV in pristine 4 ML-WSe_2_. With this, the global VBM of 4 ML-WSe_2_ at Γ̅ creates a singular Fermi surface. As the four quantized bands at Γ̅ sample the continuous *k*_*z*_-dispersive global VBM of bulk WSe_2_, the Fermi pocket is not visible for all photon energies, instead exhibiting periodic spectral weight in *k*_*z*_ (Figure 5a,d)). The valence band top is therefore visible and absent for 91.5 and 121.5 eV photons, respectively, as shown in Figure 5b,c. We note that the spacing and appearance of the four discrete W d_z^2^_ subbands at Γ̅ are slightly altered relative to free-standing 4 ML-WSe_2_ (Figure 5d–f), again likely due to interactions with the out-of-plane Nb d and Se p orbital-derived bands of the bulk substrate.

**Figure 5:**
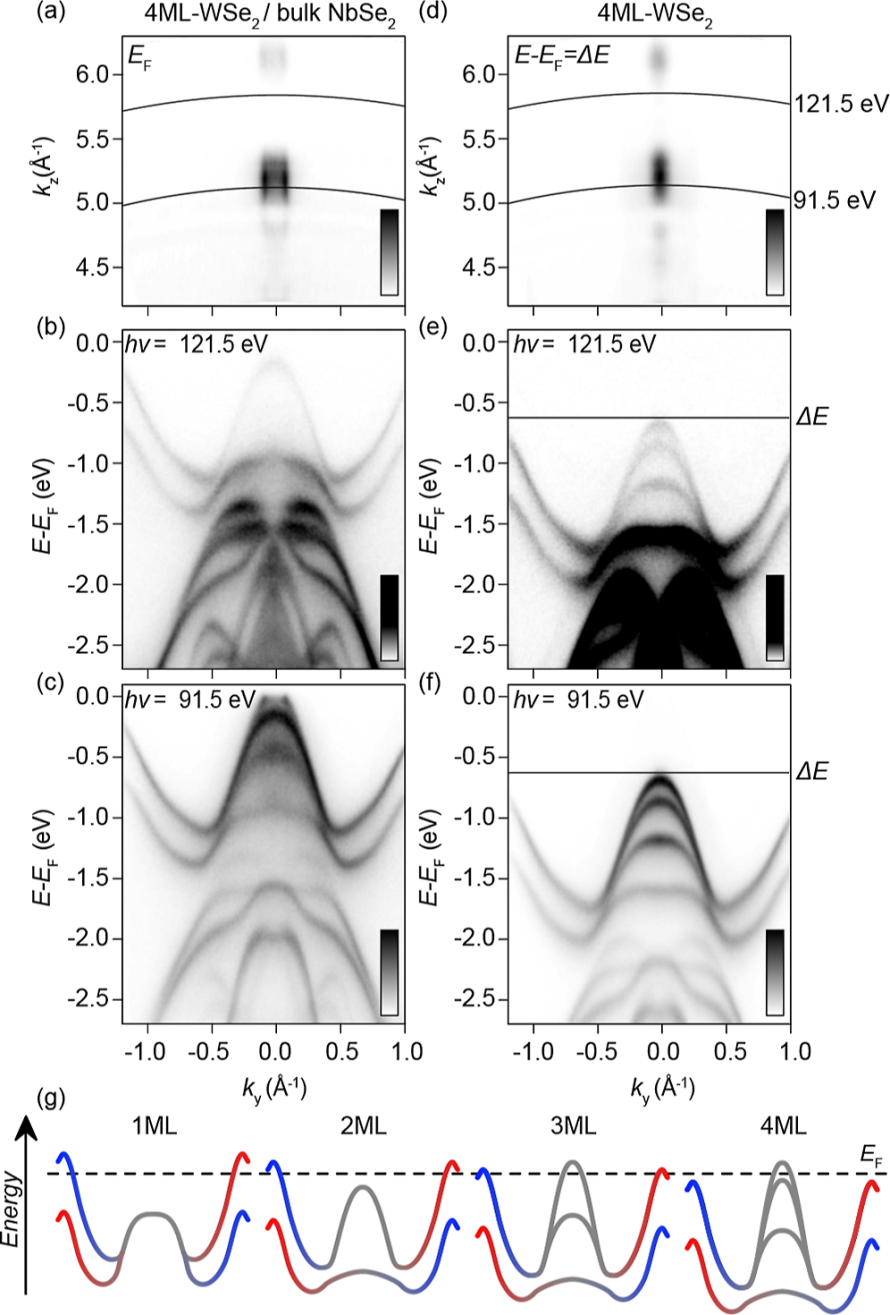
Singular Fermi surface in 4 ML-WSe_2_/bulk NbSe_2_: (a) Fermi level *k*_*y*_ – *k*_*z*_ contour showing the dimensionality of the Fermi pocket at Γ. Black lines indicate the positioning of band dispersions presented in (b,c). (b,c) Band dispersions close to the K–Γ–K axis, taken with 121.5 (b) and 91.5 eV (c) photons. (d–f) Equivalent data sets for 4 ML-WSe_2_/gr. The *k*_*y*_ – *k*_*z*_ contour in (d) is presented for *E* – *E*_F_ = Δ*E*, the calculated charge transfer when interfaced with bulk NbSe_2_. (g) Schematic illustrating the likely Fermi level placement in the surface layers for 1–4 ML-WSe_2_/bulk NbSe_2_.

The 0.63 eV energy shift of the bands in the 4 ML scenario is smaller than observed for the monolayer limit (∼0.85 eV), but the resulting Luttinger count is similar (∼10^13^ cm^–2^ in both cases when assuming that Fermi surfaces formed in the ML and 4 ML scenarios have degeneracies of 1 and 2, respectively^36^). The reduced potential difference and constant carrier density are consistent with the increased thickness of the WSe_2_ top layer and therefore the increased separation of displaced charge.

As schematized in Figure 5g, one should expect that the 2 and 3 ML scenarios produce the intermediate scenarios: the gradual shift of the global VBM from K to Γ with increasing film thickness should result in distinct Fermi surfaces from those observed in our systems, and there may exist a thickness where both Fermi surfaces exist simultaneously. A similar Γ̅-centered Fermiology was achieved recently by interfacing few-layer WSe_2_ with few-layer NbSe_2_,^37^ demonstrating how similar behavior can be achieved without a bulk component (evidenced also through the calculations for finite-thickness NbSe_2_ in Figure 4), increasing suitability for integration into devices. We note, however, that pairing WSe_2_ to few-layer NbSe_2_ will generically result in the generation of well-defined and delocalized states within the array of d_z^2^_ derived bands at Γ̅, in addition to those schematized in Figure 5g, potentially polluting surface transport signals from K/K′.^37^

## Conclusions

In conclusion, we have demonstrated that matching thin flakes of WSe_2_ to many-layer NbSe_2_ single crystals creates p-type contacts with negative Schottky barriers, and the momenta and spin degeneracy of the charge carriers at the interface can be tuned with the thickness of the WSe_2_ overlay.

For the case of monolayer WSe_2_, the Fermi level is shifted between the local band maximum at Γ and the global valence band maximum at K. While the band at Γ becomes resonant with out-of-plane orbitals from NbSe_2_ and hence delocalized into the bulk, the K′ and K point valleys remain localized to the WSe_2_ monolayer due to their in-plane orbital makeup. This observed localization alongside our density functional theory calculations together implies that the spin-valley locking of free-standing WSe_2_ monolayers is preserved. The hybrid WSe_2_/NbSe_2_ system thus behaves analogously to a bulk solid wherein Rashba-split surface states cross the Fermi level, but with out-of-plane spin polarization and a maximized momentum separation.

The semimetallic equilibrium state found here is likely further tunable through gating, and the relative size of the spin-polarized hole pockets at K and K′ could be potentially changed with, e.g., uniaxial strain or applied electric fields. It is noteworthy that 2H–NbSe_2_ possesses competing charge density wave (CDW) and superconducting phases, which are strongly thickness dependent (transition temperatures of ∼34 and 7 K, respectively, in the bulk limit).^38–41^ While the twist angle between the WSe_2_ monolayer and bulk NbSe_2_ is large in this proof-of-principle example, other twist angles could lead to a pronounced Moiré periodicity, which could be sensitive to the CDW reconstruction of the substrate.^42^ More excitingly, the superconducting phases of NbSe_2_ could lead to odd-parity pairing between the spin-polarized Fermi pockets, a scenario which is thought to be one pathway toward topological superconductivity.^43^ More generally, this work bolsters the collective electronic properties of interfaced TMD systems^23,24,37,44–47^ while providing spectroscopic evidence for electronically ohmic contacts with negative Schottky barriers between a TMD semiconductor and metal, a result of technological importance for the ultimate optimization and miniaturization of electronic devices.

## Methods/Experimental Section

### Heterostructure Fabrication

The photoemission spectra from bulk NbSe_2_ in Figure 1b and Supplementary Figure 2 originate from high-quality single crystals of NbSe_2_, cleaved *in situ* in measurement conditions with a standard top post method. All TMD flakes were exfoliated onto polydimethylsiloxane (PDMS) using blue tape, and their thicknesses were verified through photoluminescence spectroscopy (e.g., Figure 1d, performed with a diode-pumped solid-state CW 532 nm laser, focused with a 100× objective on the flake of interest) and/or by comparing optical images and photoemission spectra to those of known references. The ML-WSe_2_/bulk NbSe_2_ heterostructure characterized in Figures 2 and 3 and Supplementary Figure 1 is created through the transfer of a monolayer WSe_2_ from PDMS to a freshly cleaved bulk NbSe_2_ crystal in an Ar atmosphere. The sample is then rinsed with diisopropylamine, isopropanol, and ethanol and annealed in UHV at 200 °C for 3 + h prior to photoemission experiments. For the 4 ML-WSe_2_/bulk NbSe_2_ sample in Figure 5, a many-layer flake of NbSe_2_ was transferred from PDMS onto a graphite/*n*-type Si substrate within an Ar glovebox. Following chemical rinsing and annealing, the 4 ML region of WSe_2_ was then transferred on top. The sample is then rinsed again and annealed in UHV at 200 °C for 3+ hours prior to photoemission experiments. The ‘free-standing’ flakes of WSe_2_ shown in Figures 1, 2, and 5 were prepared in one of two different ways: the 1 and 4 ML WSe_2_ flakes in Figure 1a and Figure 5d–f, respectively, were transferred in air from PDMS onto graphite/*n*-type Si substrates. After a chemical rinse, the samples were annealed in UHV at 270 °C for 3 + h prior to photoemission. The role of the graphite is to prevent the WSe_2_ flake from being lost during the chemical rinsing process. The ML flake in Figure 2c was transferred in air onto a *h*BN/undoped Si substrate and grounded via graphene to Au contacts. The sample was chemically rinsed and annealed in a constant-flow Ar furnace at 100 °C for 1 h after each stacking stage and annealed in UHV at 270 °C prior to photoemission.

### Photoemission

Photoemission data were acquired from one of three sources: (1) A nano-ESCA system with a helium lamp (NanoESCA III at FMMA (ROR: 04z91ja70)^48^) at room temperature was used for the spectra in Figure 1a. (2) Nanofocused angle-resolved photoemission (nano-ARPES) (∼2 μm light spot) at the MAESTRO beamline of the Advanced Light Source (LBNL) was used for the data in Figures 2 and 5 and Supplementary Figure 1, acquired at cryogenic temperatures of ∼25 K with *p*-polarized photons of energies between 60 and 150 eV. (3) The i05 beamline of Diamond Light Source was utilized for the data in Figure 1b and Supplementary Figure 2, acquired at ∼10 K with *p*-polarized photons of between 106 and 121 eV. Mapping from photon energy to *k*_*z*_ was done using the free-electron final state assumption,^21^ assuming an inner potential of 13 eV to match that found for bulk WSe_2_.^8^

### Theory

Electronic structure calculations were performed within density functional theory using the Perdew–Burke–Ernzerhof exchange–correlation functional,^49^ as implemented in the Quantum ESPRESSO package.^50,51^ We used the norm-conserving pseudopotentials^52^ and plane-wave basis set with a cutoff energy of 70 Ry. Due to the close similarity between the lattice parameters of 2H–WSe_2_ and 2H–NbSe_2_, the experimentally reported bulk structure of 2H–WSe_2_ ^53^ was used for both compounds in constructing WSe_2_/NbSe_2_ heterostructures. A unit cell containing 1 ML WSe_2_ and five-layer NbSe_2_ with a zero twist angle was employed, with a vacuum spacing of 40 Å to prevent spurious interactions between periodic images. The Brillouin zone was sampled using a *k*–point mesh of 12 × 12 × 1. Spin and orbital projections were obtained using Wannier functions,^54,55^ downfolded from the DFT Hamiltonian, with the valence d orbitals of the transition–metal atoms and the Se 4p orbitals chosen as projection centers.

## Supplementary Material



## Data Availability

Data are available from the authors upon reasonable request.

## Notes

Supplemental data sets demonstrating homogeneity of the 1 ML-WSe_2_/NbSe_2_ interface and reference data for bulk NbSe_2_ (PDF)
