# Supplementary material for: Negative Schottky Barriers and Spin-Polarized Fermi Crossings at WSe2/NbSe2 Interfaces
Source: ACS Nano. 2026 Feb 25;20(9):7898–907. doi: 10.1021/acsnano.5c22009 (PMC12981010; doi:10.1021/acsnano.5c22009)
Supplement: Supplementary file 1 [file nn5c22009_si_001.pdf]

Supporting Information for: Negative Schottky barriers and spin-polarised Fermi crossings at $WSe_2/NbSe_2$ interfaces

Oliver J. Clark,[1,2,*] Anugrah Azhar,[3,4] Thi-Hai-Yen Vu,[1] Benjamin A. Chambers,[5] Federico Mazzola,[6] Sadhana Sridhar,[1] Geetha Balakrishnan,[7] Aaron Bostwick,[8] Chris Jozwiak,[8] Eli Rotenberg,[8] Sarah L. Harmer,[9,5] Mohammad Saeed Bahramy,[3] Michael S. Fuhrer,[1,10] and Mark T. Edmonds[1,10,11]

[1]*School of Physics and Astronomy, Monash University, Clayton, VIC 3168, Australia*
[2]*Diamond Light Source, Harwell Science and Innovation Campus, Didcot, OX11 0DE, UK.*
[3]*Department of Physics and Astronomy, University of Manchester, Oxford Road, Manchester M13 9PL, UK*
[4]*Physics Study Program, Faculty of Science and Technology, Syarif Hidayatullah State Islamic University Jakarta, Tangerang Selatan 15412, Indonesia*
[5]*Flinders Microscopy and Microanalysis, Flinders University, Adelaide, South Australia 5042, Australia*
[6]*Department of Physics and Astronomy 'Galileo Galilei', University of Padova, Padova, Italy*
[7]*Department of Physics, University of Warwick, Coventry CV4 7AL, United Kingdom*
[8]*Advanced Light Source, Lawrence Berkeley National Laboratory, Berkeley, CA, 94720 USA*
[9]*Institute for Nanoscale Science and Technology, Flinders University, Adelaide, South Australia 5042, Australia*
[10]*ARC Centre for Future Low Energy Electronics Technologies, Monash University, Clayton, VIC 3168, Australia*
[11]*ANFF-VIC Technology Fellow, Melbourne Centre for Nanofabrication, Victorian Node of the Australian National Fabrication Facility, Clayton, VIC 3168, Australia*

Homogeneity of overlap regions

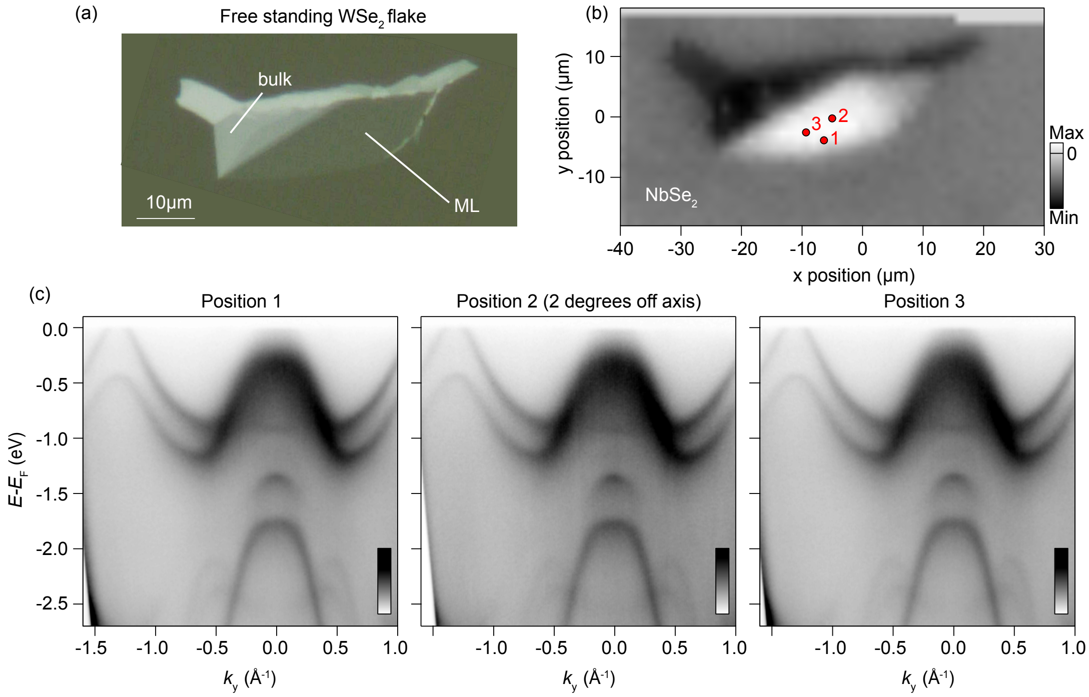

FIG. 1. Homogeneity of interfaced sample regions (a) Optical image of the exfoliated WSe_2 flake prior to interfacing with bulk $NbSe_2$. The monolayer (ML) region is indicated. (b) Image of the interface region of ML-WSe_2/bulk $NbSe_2$ made from scanning the valence band region as a function of spatial position using 91.5 eV photons. The colour contrast at each spatial position is the result of subtracting the integrated region of electronic structure below ∼-1.25 eV from that of the near-E_F electronic structure. This highlights the sample regions with bands near the Fermi level, thus clearly distinguishing p-doped regions from other sample areas. Red circles indicate the positions of high-statistics K'-Γ-K band dispersions in (c). (c) K'-Γ-K band dispersions at the spatial positions indicated in (b). The spectrum at position 2 has a slight azimuthal misalignment.

In Supplemental Figure 1(a-b), we compare optical images of the free-standing WSe_2 flake (prior to interfacing to $NbSe_2$) to a map constructed during nano-ARPES measurements by spatially varying the sample position while collecting valence band spectra (after interfacing to $NbSe_2$). The intensity at each pixel is the result of subtracting the integrated region of electronic structure below ∼1.25 eV binding energy from that of the near-E_F electronic structure region. By doing this, the sample region corresponding to the monolayer WSe_2 is very clearly identified. Moreover, the intensity is uniform over the majority of the sample region, indicating that there are no local-scale variations of p-doping at the interface.

In Supplemental Figure 1(c), spectra from three different spatial positions within the monolayer overlap region are compared. There are negligible differences between the spectra, again indicating a homogeneous interface.

Reference datasets for bulk $NbSe_2$

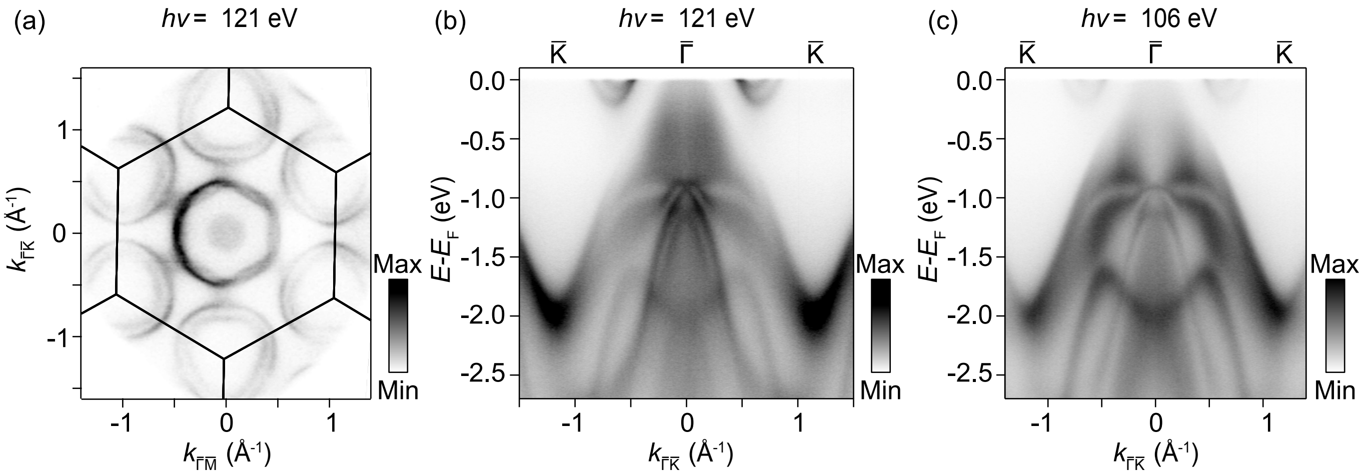

FIG. 2. Electronic structure of bulk 2H-$NbSe_2$ by ARPES (a) Fermi surface map ($h\nu$=121 eV) of $NbSe_2$, for comparison to the datasets in Figure 4 of the main text. The surface Brillouin zone of $NbSe_2$ is overlaid in black. (b,c) $\overline{\mathrm{K}}$'-$\overline{\Gamma}$-$\overline{\mathrm{K}}$ band dispersions for $h\nu$=121 eV (b) and $h\nu$=106 eV (c). Note the presence of an intense and sharp band crossing at $E - E_\mathrm{F}$ ∼-0.9 eV in both cases.

* Corresponding author. E-mail address: oliver.clark@diamond.ac.uk
